# GeneMANIA update 2018

**DOI:** 10.1093/nar/gky311

**Published:** 2018-06-15

**Authors:** Max Franz, Harold Rodriguez, Christian Lopes, Khalid Zuberi, Jason Montojo, Gary D Bader, Quaid Morris

**Affiliations:** 1 The Donnelly Centre, University of Toronto, Ontario, Canada; 2 Department of Computer Science, University of Toronto, Ontario, Canada; 3 Department of Molecular Genetics, University of Toronto, Ontario, Canada; 4 Department of Electrical and Computer Engineering, University of Toronto, Ontario, Canada

## Abstract

GeneMANIA (http://genemania.org) is a flexible user-friendly web site for generating hypotheses about gene function, analyzing gene lists and prioritizing genes for functional assays. Given a query gene list, GeneMANIA finds functionally similar genes using a wealth of genomics and proteomics data. In this mode, it weights each functional genomic dataset according to its predictive value for the query. Another use of GeneMANIA is gene function prediction. Given a single query gene, GeneMANIA finds genes likely to share function with it based on their interactions with it. Enriched Gene Ontology categories among this set can point to the function of the gene. Nine organisms are currently supported (*Arabidopsis thaliana, Caenorhabditis elegans, Danio rerio, Drosophila melanogaster, Escherichia coli, Homo sapiens, Mus musculus, Rattus norvegicus* and *Saccharomyces cerevisiae*). Hundreds of data sets and hundreds of millions of interactions have been collected from GEO, BioGRID, IRefIndex and I2D, as well as organism-specific functional genomics data sets. Users can customize their search by selecting specific data sets to query and by uploading their own data sets to analyze. We have recently updated the user interface to GeneMANIA to make it more intuitive and make more efficient use of visual space. GeneMANIA can now be used effectively on a variety of devices.

## INTRODUCTION

The GeneMANIA prediction server (http://genemania.org) has been in operation for seven years (1). There have been two previous publications describing the server, its features and its prediction accuracy (1,2). Technical details on the label propagation algorithms used by GeneMANIA to find related genes (3–5) and the algorithm used to assign weights to networks (3,6) have been published separately. The prediction server functionality is mirrored by a Cytoscape app (7) for desktop use, and the function prediction algorithms are available as a set of software tools that can be run from the command line for large-scale automation. All of GeneMANIA’s data are available for download from our webserver (http://genemania.org/data) and via web services through our Cytoscape app (7).

In this article, we describe updates to the GeneMANIA prediction server, Cytoscape app, and data sets that have occurred over the past five years.

### Use cases

GeneMANIA provides a very flexible interface to query genomic, proteomic, and gene function data. It addresses three main use cases: single gene queries, multiple gene queries and network search.

Given a single query gene, GeneMANIA will find the most closely connected genes among the networks and attributes selected by the user. By default, GeneMANIA weights these data using patterns of gene co-annotation in the Gene Ontology biological function hierarchy. When the query gene is poorly characterized, hypotheses about its function can be derived from the results of GeneMANIA’s gene enrichment analysis of its connected genes (8).

Given a longer query list of genes, GeneMANIA will find more genes like those in the list. By default, with a long enough list (currently five or more genes), GeneMANIA will weight data sources based on their predictive value for reconstructing the query list. For example, given query genes with similar protein domain structures, GeneMANIA will highly weight protein domain similarity networks and will suggest more genes with a similar domain structure. Given query genes that are part of a protein complex, GeneMANIA will often find additional members of that complex and give high weight to physical interactions or predicted physical interactions – e.g. from orthologous interactions provided by I2D (9)). Heterogeneous gene lists may lead to multiple tightly connected clusters of genes with similar function, especially when links from co-expression networks are removed. To minimize browser performance issues, we only allow up to 100 genes and 100 attributes on the web app, our Cytoscape app (7) removes this restriction.

Finally, GeneMANIA is a general purpose network search engine that permits the user to select specific sets of networks out of hundreds of options. GeneMANIA will return the connections among query genes. Note that users should select one of the ‘equal weighting’ network weighting algorithms if they want to view all connections, as other weighting methods can assign a weight of zero to some of the selected networks. This mode is particularly useful when used in the Cytoscape app to find a network of interactions to process further using Cytoscape’s analysis features.

## UPDATES

### New organisms and data sources

We added support for two model organisms since 2013, *Danio rerio* (zebrafish) (10) and *Escherichia coli* (11). For zebrafish, we have 53 networks with just over 8 million interactions. For *E. coli*, we have just over 850 000 interactions spanning 24 networks.

As of 13 March 2017, GeneMANIA has almost 2300 networks that collectively contain nearly 600 million interactions covering almost 164 000 genes. Data sources with a small total number of interactions may be joined into ‘combined’ networks. We also support 28 attribute groups, which contain nearly 50 000 attributes (see Table 1). A gene attribute is an annotation for the gene. For instance, an attribute of TP53 is that it is annotated in the ‘PLK3 signalling events’ pathway.

### User interface

**Figure 1.**
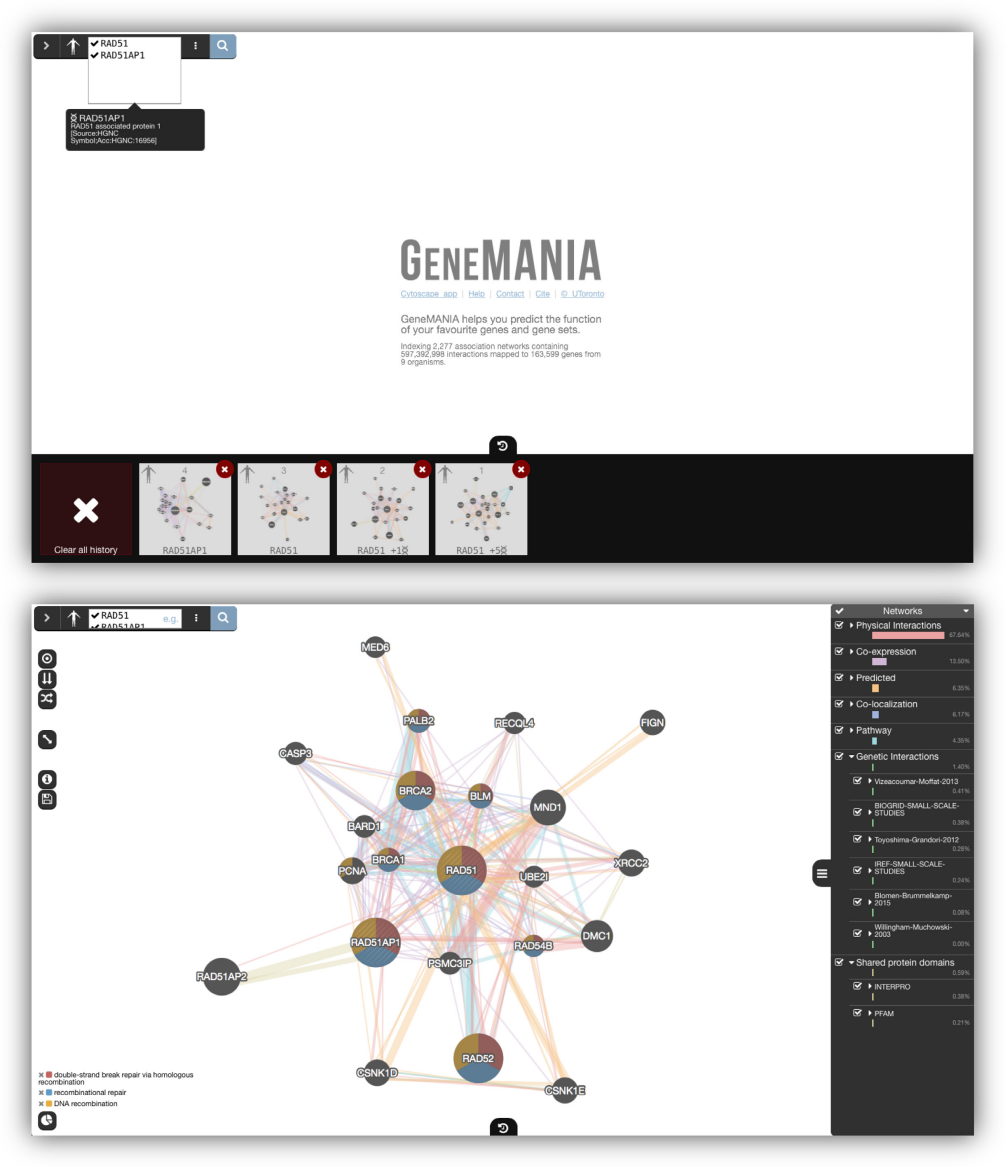
The initial GeneMANIA query view (top) and the result view (bottom). The new query history feature is shown at the bottom of query view.

We have redesigned the user interface of GeneMANIA and added a number of features (see Figure 1). The app has a new, minimalistic design that focusses on the full-screen, interactive network visualization—which is powered by Cytoscape.js (14). The apps’s main features can be accessed simultaneously on one screen, increasing information density and usability. For example, the gene list, network list, and function list can be viewed simultaneously. We now make use of icons rather than text where possible to conserve screen real-estate and to better accommodate users whose first language is not English. We carried out the redesign with support for touch devices, such as tablets. As a whole, the redesign allows users to access GeneMANIA’s features more easily and efficiently.

#### Query interface

The query interface has been condensed. Whereas it previously dominated the top of the screen, it is now contained in a small, collapsible bar on the top-left of the interface. Gene input has a quick, spell check interface: Unrecognised gene names are underlined in red. A preview is shown when the user types a gene name to help the user verify his or her entry.

#### Network layout

**Figure 2.**
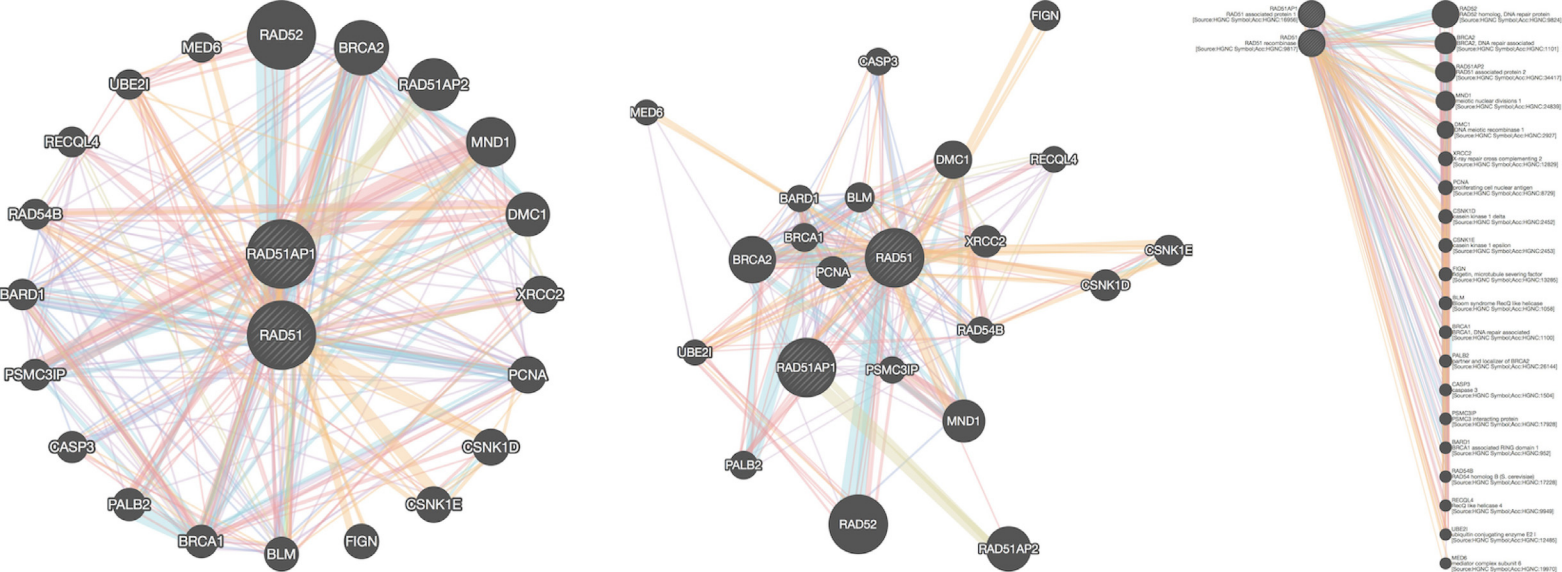
Layouts supported by GeneMANIA, from left to right: concentric bipartite, force-directed, linear bipartite.

Several layouts are supported. Each layout lets the user view the network from a different perspective (see Figure 2). Layouts are animated to provide a smooth transition from the current network view to the new one after a layout is run. The user has the option to undo the result of a layout.

The default, force-directed CoSE layout (15) orients the network such that the natural topology of the graph is evident.

The concentric circle layout gives a condensed bipartite view of the network, with query genes in the inner circle and genes GeneMANIA found in the outer circle. Genes are ordered by score, i.e. relatedness to the query gene list, in bipartite layouts.

The linear layout gives a detailed bipartite view of the network that makes it easy to see interactions between query genes and non-query genes. Gene descriptions appear in line. This layout replaces the genes tab from the previous version. This allows for viewing gene information at the same time as the network list, which was not possible before.

Attribute nodes are shown as an additional concentric circle or column in the concentric or linear layouts, respectively.

#### Gene function

GeneMANIA allows the user to colour the network nodes by function (GO annotation). The new user interface allows for up to seven simultaneous colourings. Previously, colourings were stacked and only the topmost matching colour in the stack would be painted on a gene. A function legend is now shown on the bottom-left of the screen.

#### Gestures

We added improved support for gestures and shortcut keys. Graph viewport operations support standard gestures across input devices. For example, the network can be panned by clicking and dragging with a mouse or by swiping with one finger on a touch-screen. We also improved gene highlighting: A gene’s neighbourhood is highlighted when hovering over or tap-holding on a gene, making it much easier to focus on particular sets of interactions. Refer to Table 2 for the full list of shortcuts and gestures.

**Table 1.** Attribute groups supported by GeneMANIA

Organism	Attribute group
*A. thaliana*	Interpro: protein domain attributes
*C. elegans*	Interpro: protein domain attributes
*D. rerio*	ZFIN: developmental timing expression
	Ensembl: protein family attributes
	InterPro: protein domain attributes
	KEGG: pathway annotations
	BioMart: orthologs in other species
	PFAM: protein family attributes
	Musso *et al.*: phenotype predictions (10)
	Superfamily: associated Superfamily identifiers (12)
*D. melanogaster*	InterPro: protein domain attributes
*E. coli*	InterPro: protein domain attributes
*H. sapiens*	Enrichment map: consolidated pathways 2013 (13)
	Enrichment map: drug interactions 2013 (13)
	InterPro: protein domain attributes
	Enrichment map: miRNA target predictions from MSigDB (13)
	Enrichment map: transcription factor targets from MSigDB (13)
*M. musculus*	InterPro: protein domain attributes
*R. norvegicus*	InterPro: protein domain attributes
*S. cerevisiae*	InterPro: protein domain attributes

**Table 2.** Gestures and shortcuts supported by GeneMANIA

Action	Gestures and shortcuts
Pan	Click and drag background, swipe background, arrow keys
Zoom	Mouse wheel, pinch-to-zoom, ‘+’ and ‘-’ keys
Highlight gene’s neighbourhood	Hover over a gene, tap-hold on a gene
Concentric layout	‘J’ key
Linear layout	‘K’ key
Force-directed layout	‘L’ key
Fit to screen	‘F’ key
Quick edit query genes	‘/’ key
Quick submit query	‘alt’, ‘ctrl’, or ‘command’ key with ‘enter’ when inside the gene entry box

#### Query history

We added the ability to see the list of queries that a user has made within the app. This history is available as a collapsible panel at the bottom of the screen (see Figure 1). Each query is represented by a screen-shot of the query result, with a summary of the input organism and genes. A query in the history can be clicked to reload it in the interface.

#### Report

We revised the report generation functionality with improved formatting and readability, including a much larger figure of the network. GeneMANIA directly generates PDF-formatted reports now, whereas previously the user was required to rely on the print-to-PDF browser feature.

### Cytoscape app integration

GeneMANIA has an app, previously referred to as a plugin, to integrate with the Cytoscape desktop software. The app has been updated to enable live GeneMANIA web queries through Cytosape’s new Network Search Bar. The user selects an organism and enters one or more gene names, Cytoscape makes a query to GeneMANIA’s web services, and then the resultant GeneMANIA network is displayed. Any of Cytoscape’s analysis features can then be used on this network. This provides a quick, one-step method of accessing GeneMANIA in Cytoscape.

The GeneMANIA Cytoscape app has also been updated to support CyREST, a facility for scripting with Cytoscape. The user makes REST (HTTP) calls to access or manipulate GeneMANIA within Cytoscape. This enables workflow automation for users from multiple scripting languages, and it enables other Cytoscape apps to make GeneMANIA queries.

## OUTLOOK

We plan to continue to make improvements to the GeneMANIA ecosystem, chief among them are additions to and refreshes of the source data.

We currently have a beta version of GeneMANIA webservices, which are currently being used by the GeneMANIA Cytoscape app. Future versions of the webservices will be simplified and documented for easier client consumption. Also within Cytoscape, GeneMANIA services are being integrated with the Enrichment Map pathway analysis workflow (13).

On the user interface side, a search feature for networks will be added to the web app. This will enable the user to find and toggle networks based on criteria such as the network name or category, giving the user the opportunity to explore GeneMANIA results for particular data sources more easily. Support for searching based on gene groups will allow for a user to specify topics of interest. For example, a user could enter ‘breast cancer’ to do a query with genes known to be related to breast cancer without the user having to manually specify the list.
